# Differential Mnemonic Contributions of Cortical Representations during Encoding and Retrieval

**DOI:** 10.1162/jocn_a_02227

**Published:** 2024-10-01

**Authors:** Cortney M. Howard, Shenyang Huang, Mariam Hovhannisyan, Roberto Cabeza, Simon W. Davis

**Affiliations:** Duke University; University of Arizona

## Abstract

Several recent fMRI studies of episodic and working memory representations converge on the finding that visual information is most strongly represented in occipito-temporal cortex during the encoding phase but in parietal regions during the retrieval phase. It has been suggested that this location shift reflects a change in the content of representations, from predominantly visual during encoding to primarily semantic during retrieval. Yet, direct evidence on the nature of encoding and retrieval representations is lacking. It is also unclear how the representations mediating the encoding–retrieval shift contribute to memory performance. To investigate these two issues, in the current fMRI study, participants encoded pictures (e.g., picture of a cardinal) and later performed a word recognition test (e.g., word “cardinal”). Representational similarity analyses examined how visual (e.g., red color) and semantic representations (e.g., what cardinals eat) support successful encoding and retrieval. These analyses revealed two novel findings. First, successful memory was associated with representational changes in cortical location (from occipito-temporal at encoding to parietal at retrieval) but not with changes in representational content (visual vs. semantic). Thus, the representational encoding–retrieval shift cannot be easily attributed to a change in the nature of representations. Second, in parietal regions, stronger representations predicted encoding failure but retrieval success. This encoding–retrieval “flip” in representations mimics the one previously reported in univariate activation studies. In summary, by answering important questions regarding the content and contributions to the performance of the representations mediating the encoding–retrieval shift, our findings clarify the neural mechanisms of this intriguing phenomenon.

## INTRODUCTION

Increasingly, neuroimaging studies of episodic memory investigate the relationship between memory processes and representations during memory encoding and retrieval. One key finding in these studies is the observation of overlaps in the brain regions that exhibit similar activation patterns during both the encoding and retrieval phases of memory processing. This phenomenon is referred to as “encoding–retrieval similarity” (or typically ERS). In other words, the neural response patterns engaged when a memory was initially formed (during encoding) are detected when that memory is later recalled or retrieved. This finding supports the idea that memories are stored in a distributed manner across the brain, and the neural patterns representing those memories can be reinstated during retrieval (Riegel et al., 2022; Wing, Ritchey, & Cabeza, 2015; Ritchey, Wing, LaBar, & Cabeza, 2013). At the same time, there is evidence of differences in representation between the two phases, such as robust representations of visual stimuli in occipito-temporal cortex during encoding but in lateral parietal cortex during retrieval (Long & Kuhl, 2021; Favila, Samide, Sweigart, & Kuhl, 2018; Xiao et al., 2017). Although this phenomenon, which we term *representational encoding–retrieval shift* (or RERS), has been replicated several times, several questions remain unanswered.

First, it is unclear whether the RERS involves a substantive alteration in the fundamental nature of information representations in the brain. Notably, some researchers have put forth the notion that these representations undergo a transformation, transitioning from a sensory/concrete format during the encoding phase to a more semantic/abstract format during the retrieval phase (Favila, Lee, & Kuhl, 2020). This idea is intuitive, considering that during encoding, there is typically an external visual input, whereas during retrieval, memories are at least partly reconstructed through a semantically guided generation process (Brown & Craik, 2000). However, there is also evidence that occipito-temporal representations during encoding can be semantic and abstract (Davis et al., 2021). Moreover, representations during retrieval are not consistently characterized by a purely abstract nature; at times, they exhibit detailed, sensory-like qualities (Vo et al., 2022; Brady, Konkle, Gill, Oliva, & Alvarez, 2013). To address this issue, it is necessary to employ analyses that can distinguish between different types of visual and semantic representations, an approach not utilized in previous studies reporting RERS. Model-based representational similarity analysis (RSA) emerges as a potent tool in this context. RSA involves establishing connections between multivoxel brain activation patterns and theoretical models of stimulus features, encompassing both sensory and semantic models (Davis et al., 2021; Kriegeskorte & Kievit, 2013) and can determine the strength of the corresponding representation. RSA can, therefore, address whether encoding and retrieval representations shift along a sensory-semantic dimension.

Second, although RERS has been observed during memory tasks (Long & Kuhl, 2021), it has not been directly linked to memory performance. Therefore, RERS could reflect any of the dissimilarities between encoding and retrieval tasks, such as differences in stimuli rather than in memory processes. Addressing this issue is crucial for determining if RERS is truly a memory phenomenon. Furthermore, linking RERS to memory performance could clarify the role of parietal representations during retrieval. In univariate fMRI studies, the phenomenon known as *encoding–retrieval flip* (Huijbers et al., 2012; Daselaar et al., 2009) refers to finding that parietal activity is associated with encoding failure (forgotten > remembered trials) but with retrieval success (remembered > forgotten trials). If parietal representations in RERS exhibit the same pattern, it is possible that similar mechanisms underlie both univariate and representational phenomena.

**Figure 1.**
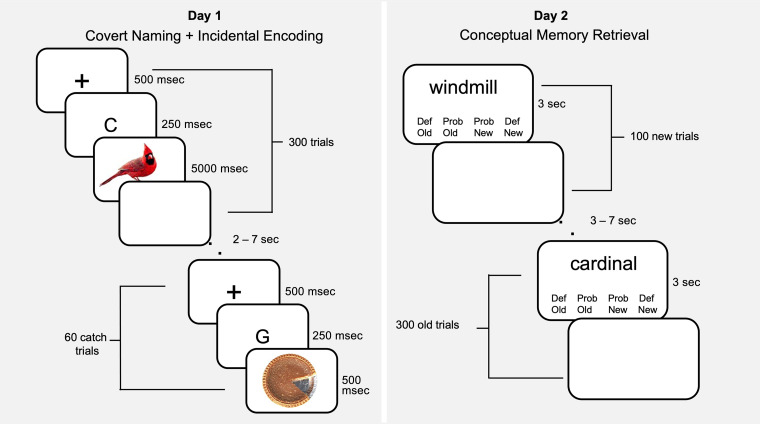
Paradigm. Left. Incidental encoding phase on Day 1. Participants covertly named 360 objects and indicated if the preceding letter did not match the image. Right. The conceptual memory test on Day 2 consisted of previously viewed and novel concepts. Participants indicated confidence in their old or new judgments with one of four button presses.

To investigate these questions, we conducted an experiment where participants encoded pictures of everyday objects and retrieved them in response to the object names (see Figure 1). The study had two primary objectives. First, using model-based RSA to differentiate visual versus semantic representations, we tested the hypothesis that representations shift from a more sensory/concrete format during encoding to a more semantic/abstract format during retrieval. Second, unlike previous RERS studies, we utilized an item-wise approach to directly compare the representations for remembered versus forgotten stimuli during encoding and retrieval. Finding that RERS occurs for successful memory representations (remembered > forgotten) would suggest that RERS is driven by memory processes. Otherwise, the results would suggest that RERS reflects some other dissimilarity (e.g., stimuli). We were particularly interested in lateral parietal cortex, as finding that parietal representations are associated with encoding failure but not with retrieval success would indicate a connection between RERS and the encoding–retrieval flip.

## METHODS

### Participants

Twenty-six healthy younger adults were recruited for this study (all native English speakers; 14 female participants; age mean = 20.4 years, *SD* = 2.4 years; range: 18–26 years) and participated for monetary compensation. This sample size is in line with previous work investigating mnemonic effects in multivariate fMRI (Ritchey et al., 2013). Informed consent was obtained from all participants under a protocol approved by the Duke Medical School institutional review board (IRB). All procedures and analyses were performed in accordance with IRB guidelines and regulations for experimental testing. Participants had no history of psychiatric or neurological disorders and were not using psychoactive drugs. Of the original participants tested, four participants were excluded because of poor performance/drowsiness during Day 1, one participant suffered a fainting episode within the magnetic resonance scanner on Day 2, and two participants were subsequently removed from the analysis because of excessive motion, leaving 19 participants in the final analysis.

### Stimuli

Stimuli used in this study were 360 objects drawn from 12 object categories, including mammals, birds, fruits, vegetables, tools, clothing items, foods, musical instruments, vehicles, furniture items, buildings, and other objects. Of these 360, 300 (an average of 25 from each category) were used as the target stimuli set, as well as 60 catch-trial items (see Behavioral Paradigm in Figure 1) evenly distributed from these 12 categories. The catch trials were included in the behavioral paradigm but not in the fMRI analyses. During the study, each object was presented alone on a white background in the center of the screen, which was 7.5° in size.

### Paradigm

As illustrated by Figure 1, the behavioral paradigm consisted of separate incidental encoding (Day 1) and retrieval (Day 2) phases in subsequent days (range = 20–28 hr); participants were naive to the subsequent memory test until after scanning was complete on Day 1. During encoding, participants were instructed to covertly name each object (e.g., “cardinal,” “pie”); we explicitly chose to use covert naming (instead of a semantic elaboration task, as is common in the episodic memory studies) given evidence that basic-level naming is an automatic process (Bauer & Just, 2017). Nonetheless, although it is typical in object naming studies to rely on covert naming (Cichy, Kriegeskorte, Jozwik, van den Bosch, & Charest, 2019; Clarke, Devereux, Randall, & Tyler, 2015), we were particularly interested in ensuring participants retrieved the correct label for each presented image, given the verbal cue to be used at in the subsequent retrieval session. To ensure the correct label was brought to mind for each image, participants were instructed to indicate with a single button press, during the blank response screen proceeding the object presentation, whether a single letter probe presented immediately before each object matched the first letter of the object's name. If the letter probe did match the first letter of the object's name, participants were instructed not to press any button. On a small proportion of “catch trials” (60 of 360 total items), letters that were not associated with any potential label for a given object were shown instead of the matching letter. This ensured that if participants did not know or could not remember the object's name, they would press the “does not match” key. Catch trials (17%) and other “does not match” trials (mean = 8%) were excluded from the analyses; as such, both recognition of the catch trials and uncertainty about an object's identity were reflected in a button press. Trials were timed, with timing parameters for each trial comprising of an initial fixation cross lasting 500 msec, followed immediately by the single letter probe for 250 msec, immediately followed by an object presented for 500 msec, followed by a blank response screen lasting between 2 and 7 sec. The object order was counterbalanced across participants.

Approximately 24 hr later, participants underwent perceptual and conceptual memory tests; this lag between encoding and retrieval testing was chosen to roughly equate perceptual and conceptual memory performance. The perceptual memory test is excluded from the analyses, as they focus exclusively on results from the scanned portions of the paradigm (for a more in-depth focus on perceptual memory performance, see Davis et al., 2021). During the in-scanner conceptual memory test, participants were shown lexical cues representing 400 concepts; 300 concepts were old objects presented during the encoding phase, and 100 were new objects. Participants responded to each concept with an old/new confidence judgment using one of four buttons (definitely old, probably old, probably new, and definitely new). Trials were timed, with timing parameters for each trial comprising a concept label and the response options presented for 3 sec, followed by a blank response screen lasting between 3 and 7 sec. The concept order was counterbalanced across participants.

### MRI Acquisition and Brain Data Preprocessing

The encoding phase and the conceptual memory test were scanned. Scanning was done in a GE MR 750 3-Tesla scanner (General Electric Magnetic Resonance 3.0 Tesla Signa Excite HD short-bore scanner, equipped with an eight-channel head coil). Coplanar functional images were acquired with an eight-channel head coil using an inverse spiral sequence with the following imaging parameters: 37 axial slices, 64 × 64 matrix, in-plane resolution 4 × 4 mm^2^, 3.8-mm slice thickness, flip angle = 77^o^, repetition time [TR] = 2000 msec, echo time = 31 msec, field of view [FOV] = 24.0 mm^2^. The diffusion-weighted imaging data set was based on a single-shot EPI sequence (TR = 1700 msec, 50 contiguous slices of 2.0-mm thickness, FOV = 256 × 256 mm^2^, matrix size = 128 × 128, voxel size = 2 × 2 × 2 mm^3^, b-value = 1000 sec/mm^2^, 36 diffusion-sensitizing directions, total scan time = ∼6 min). The anatomical MRI was acquired using a 3-D T1-weighted echo-planar sequence (68 slices, 256 × 256 matrix, in-plane resolution = 2 × 2 mm^2^, 1.9-mm slice thickness, TR = 12 msec, echo time = 5 msec, FOV = 24 cm). Scanner noise was reduced with earplugs, and head motion was minimized with foam pads. Behavioral responses were recorded with a four-key fiber optic response box (Resonance Technology), and when necessary, vision was corrected using MRI-compatible lenses that matched the distance prescription used by the participant.

Functional preprocessing and data analysis were performed using SPM12 (Wellcome Department of Cognitive Neurology) and custom MATLAB (The MathWorks) scripts. Images were corrected for slice acquisition timing, motion, and linear trend; motion correction was performed by estimating six motion parameters and regressing these out of each functional voxel using standard linear regression. Images were then temporally smoothed with a high-pass filter using a 190-sec cutoff and normalized to the Montreal Neurological Institute (MNI) stereotaxic space. White matter (WM) and cerebrospinal fluid (CSF) signals were also removed from the data, using WM/CSF masks, and regressed from the functional data using the same method as the motion parameters. Single-trial BOLD responses were analyzed using a modified general linear model (Worsley & Friston, 1995).

For each gray matter voxel, the activity estimate for each encoding and retrieval trial is as follows: All trials were estimated simultaneously in a single general linear model, using a separate regressor consisting of an impulse function convolved with a double gamma hemodynamic response function (Rissman, Gazzaley, & D'Esposito, 2004). The model included a six-parameter head motion, button presses, WM signals, and CSF signals as regressors. The resulting voxel-by-trial betas reflected the fit shape of the hemodynamic response evoked by a given trial during the encoding and retrieval phase of the procedure. Single-trial betas were then used to construct activity pattern matrices (APMs; detailed below). Brain images were visualized using the FSLeyes toolbox (fsl.fmrib.ox.ac.uk/fsl/fslwiki/FSLeyes) and SurfIce (www.nitrc.org/projects/surfice/).

### Data Analysis

**Figure 2.**
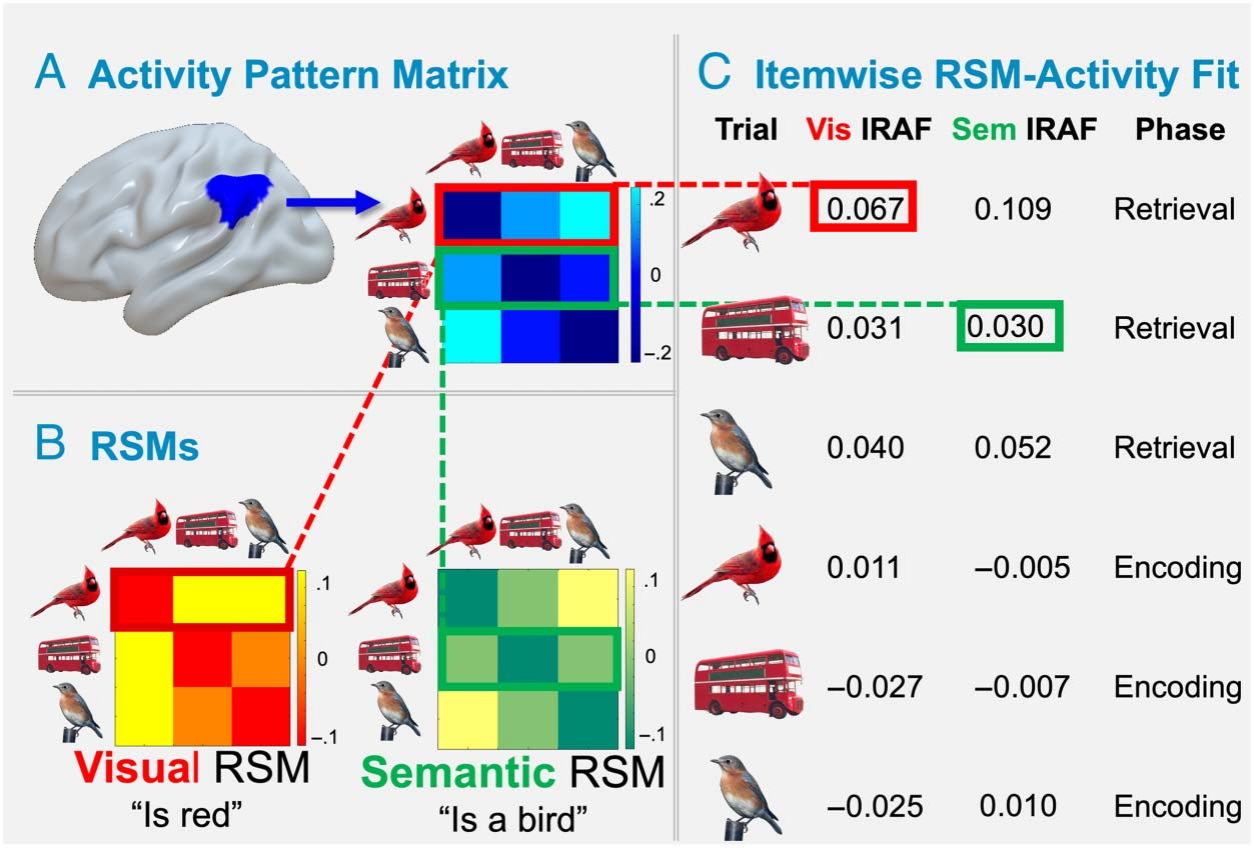
(A) Participant-specific retrieval APMs were constructed for each region in the Brainnetome Atlas. (B) RSMs were constructed for visual and semantic representation types. (C) Each item's unique retrieval activity pattern vector is correlated with that item's visual and semantic representational similarity vector, yielding a visual and semantic IRAF, respectively.

The analytical approach is visualized in Figure 2. First, to aid the investigation of changes in content in RERS, two representational similarity matrices (RSMs) of the same size were constructed using a previously reported normative study (Hovhannisyan et al., 2021). The visual RSM represented the correlations of visual features ascribed to each object. For example, a red bus is visually similar to a cardinal. The semantic RSM represented the correlations of taxonomic features (e.g., other birds are similar to cardinal in taxonomy). Next, participant-specific retrieval APMs were constructed for 72 regions along the ventral and dorsal stream (including all occipital, temporal, and posterior parietal regions) in the Brainnetome Atlas (Fan et al., 2016). The cells of the APMs represent the similarity (Pearson correlation) in fMRI activation patterns by stimuli (300 × 300 concepts presented at encoding and retrieval). Then, item-wise RSM-activity fits (IRAFs; see Davis et al., 2021) were calculated to represent the correlation between stimulus properties in the RSMs and the fMRI activity patterns in the retrieval and encoding APMs for each object (the vector on each row in the matrices). IRAF values are an item-wise index of the representational strength in a given region. This approach is a variation of traditional RSA approaches in which second-order correlations between fMRI APMs and model RSMs are computed using the entire matrix (Kriegeskorte & Kievit, 2013) but offers the advantage of capturing trial-level variance and explicitly modeling specific stimuli as a random effect. Finally, we investigated memory phase-related location and content shifts in each of the 72 regions and their contribution to memory success using a series of mixed-effect regression models.

### RSMs

To construct our RSMs, we utilized data from a previously reported study by Hovhannisyan and colleagues (2021). In this study, a comprehensive assessment was carried out on Amazon Mechanical Turk to determine the visual and semantic attributes of everyday objects, specifically using the DinoLab Object Database. This task engaged 162 self-identified American-English-speaking Amazon Mechanical Turk workers, aged between 18 and 62 years, who evaluated 946 common objects. They conducted the ratings through multiple sessions—each lasting roughly an hour and covering 30 objects. In these sessions, the workers would select a grammatical connector from a list (like “is,” “has,” or “does”) and append an attribute to the object (for instance, “a cardinal … has wings”). They were allowed to participate in up to five sessions.

During the data refinement process, several steps were taken to prepare the attribute responses for the creation of RSMs, following prior established protocols (Davis et al., 2021; Devereux, Tyler, Geertzen, & Randall, 2014; McRae, Cree, Seidenberg, & McNorgan, 2005). These procedures included removing intensifying adverbs (e.g., “very”), breaking down complex features into simpler ones (e.g., “has a round face” into “is round” and “has a face”), synonym consolidation within and across concepts to maintain consistency (e.g., different expressions of group travel merged into “does travel in groups”), spelling corrections, and morphological congruence (e.g., “is used in traveling” merged with similar expressions). In addition, any feature mentioned for only one concept was removed to maintain a generalizable feature set.

Once processed, the features were sorted into categories drawn from the McRae database (McRae et al., 2005), such as visual attributes (color, form, motion) and others like smell, sound, taste, touch, function (purpose of the object), taxonomic classification (like “is a bird”), and encyclopedic knowledge (like “lives in India”). These categories helped in classifying the features, and this classification was performed by five independent raters who demonstrated high interrater reliability (intraclass correlation coefficient > 0.8).

The product of these categorizations was feature-by-concept frequency matrix that illustrated the normalized frequency with which a given feature was reported for a given object. From this, we constructed the visual (color, form, and motion) and semantic (taxonomic) RSMs by correlating 1886 visual and 353 semantic feature attributes across each of the 300 “old” objects in our paradigm, resulting in two 300 × 300 RSMs. Each cell of the RSM indicates the similarity of the feature types ascribed to the two objects. To ensure that the visual and semantic RSMs represented sufficiently distinct features, we calculated the correlation between the entire visual and semantic matrices (rho = .23), as well as an item-wise correlation (mean rho = .27, range: −.17 to .52). Although there is some overlap between visual and semantic information, the model RSMs are not highly colinear.

### APMs

The similarity in voxel activation patterns across stimuli were created for each of the selected 72 regions in the Brainnetome Atlas by vectorizing each of the voxel level activation values and correlating them with Pearson's *r*. Whereas each cell of an RSM contains a measure of similarity in stimulus properties, each cell of an activity pattern similarity matrix (APM) contains a measure of similarity in activation patterns across stimuli. As noted above, the activation patterns were extracted for 72 ROIs. Activation patterns were then vectorized and correlated with Pearson's *r*. To minimize the effect of temporal correlation, a time RSM was constructed by calculating the closeness in time of each trial within a run and scaling it between 1 (same time) and 0 (furthest in time within a run). The time RSM was then regressed from the APM. This step was necessary to reduce the effects of temporal correlation on second-level analyses, as excluding within-run comparisons was not feasible because of the encoding phase comprising only two runs.

After correcting the AMPs for temporal correlation, the final APM excluded some trials. During encoding, excluded trials were “catch trials” (60 of 360 total items) for which the proceeding letter did not match the concept label. Trials excluded from encoding and retrieval APMs were trials for which participants could not remember the object's name and pressed the “do not know” key during the encoding phase (mean = 8% of trials).

### Phase-dependent Representational Shifts Supporting Conceptual Memory

**Figure A1.**
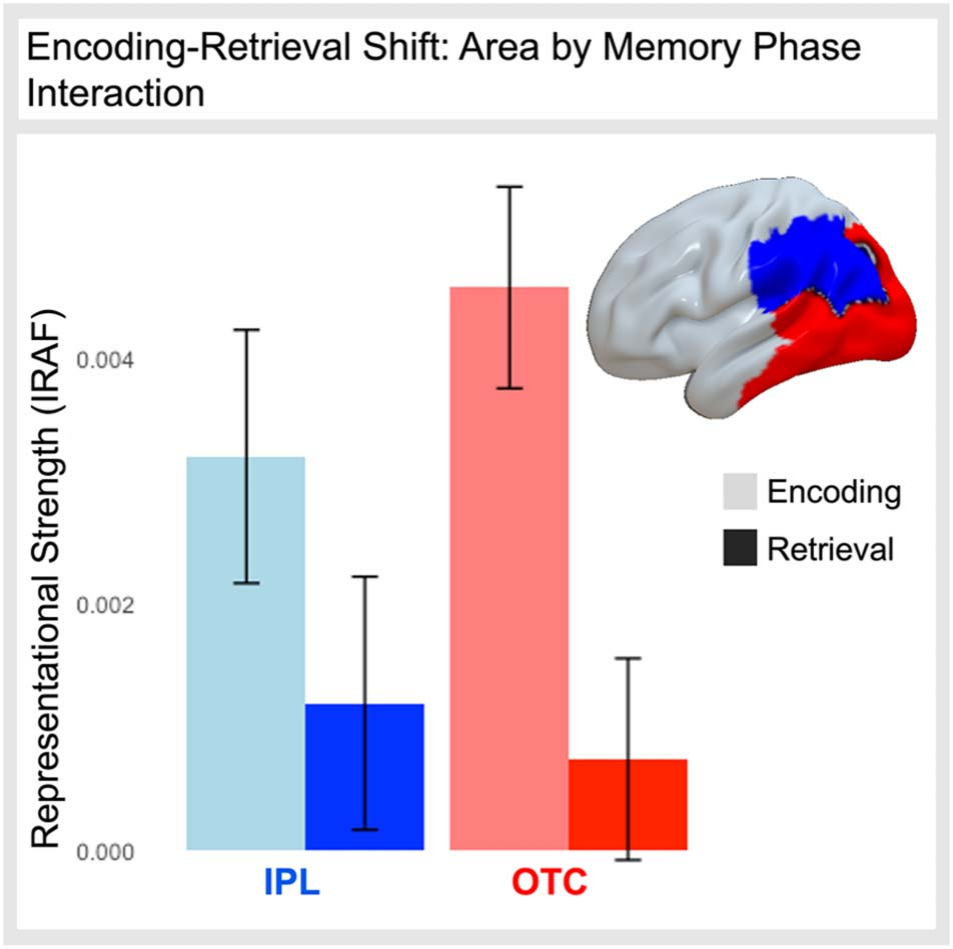
Memory Phase × ROI Type interaction. *y* axis: Representational strength (IRAF) as estimated from the mixed-effects model. ROI types are indicated on the *x* axis and brain rendering with the inferior parietal lobe in blue and the occipito-temporal cortex in red. Legend: Encoding in lighter colors, retrieval in bolder colors.

Trial-wise estimates of the semantic and visual representations during the encoding and retrieval task phases form the basis of the proceeding analyses. Descriptive estimates of such representational strength were assessed in 72 regions in the ventral and dorsal streams from the Brainnetome Atlas (Fan et al., 2016) and considered visual and semantic IRAFs, which afford the advantage of estimating trial-level variance to explicitly model specific stimuli as a random effect. To test if we replicate previous RERS findings, IRAF values for remembered trials from the 12 inferior parietal cortex (IPC) regions and 32 occipitotemporal cortex (OTC) regions (Appendix Figure A1) were submitted to a mixed-effects model testing the interaction of memory phase (encoding vs. retrieval) and area (IPC, OTC) with random intercepts of feature type, subject, stimulus, and ROI.

Because the IRAF approach allows us to index brain-model relations at an item level, we can subsequently model memory success for each trial—in contrast to approaches where successful and unsuccessful encoding is modeled as a uniform condition (Oedekoven, Keidel, Berens, & Bird, 2017). In addition, this approach accounts for an unequal number of trials for remembered and forgotten items by modeling the individual variances in memory success, as well as fitting linear mixed-effect models accounting for variance because of stimuli intercepts.

To investigate the representational content in RERS and the extent to which they support conceptual memory, IRAF values were submitted to a series of mixed-effects linear regression models using LMER4 (Bates, Mächler, Bolker, & Walker, 2015) in R (R Core Team, 2020). Models were fit using restricted maximum likelihood methods.

To investigate the representational content in RERS and the extent to which they support conceptual memory, IRAF values were submitted to a series of mixed-effects linear regression models using lme4 (Bates et al., 2015) in R (R Core Team, 2020). Models were fit using restricted maximum likelihood methods.

The model structure utilized for each ROI included fixed effects of memory success (remembered vs. forgotten), memory phase (encoding vs. retrieval), and representation type (visual vs. semantic), as well as their interactions, including the three-way interaction. Models failed to converge for eight bilateral anterior temporal regions of the 72 ROIs, leaving 65 ROIs. To evaluate the significance of fixed effects in the regression models, we used the lmerTest package (Kuznetsova, Brockhoff, & Christensen, 2017). We estimated denominator degrees of freedom using Satterthwaite's method (Satterthwaite, 1946) and conducted tests of fixed effects using *F* tests on Type III sums of squares with an alpha value of .05 false discovery rate (FDR) corrected (accounting for all the multiple comparisons). Post hoc analyses of significant interaction terms were conducted using the *diffmeans* function in the lmerTest package. All factors of interest included in the model were categorical in nature, so marginal means were computed for each level of factors in the interaction term.

To ensure sufficient statistical power, we conducted a power analysis for the estimation of linear mixed-effects models using the simr package in R (Green & MacLeod, 2016). We estimated our power to observe effects of memory success (remembered vs. forgotten), memory phase (encoding vs. retrieval), and representation type (visual vs. semantic) and their interactions on the IRAF values given an alpha level of .05 and a small-to-medium effect size of *d* = 0.4. This effect size is in line with memory success effects observed in previous representational similarity analyses (Naspi, Stensholt, Karlsson, Monge, & Cabeza, 2023). The model determined our ∼20,000 observations (19 participants, ∼300 objects, two memory phases, and two feature types) provide > 85% power to detect the effects of interest. This model, iterated over 1000 simulations and showed that 157 observations for each of the 19 participants would be sufficient; empirical models presented below rely on ∼300 objects per participant in each memory phase for each representation type, suggesting we are well powered to observe these effects.

## RESULTS

### Behavioral Responses

Participant responses consisted of conceptual retrieval response types for covertly named trials during encoding. “Probably” and “definitely” responses to old items were collapsed because of the relatively low number of “probably old” responses (mean = 52). Some participants exhibited a response bias for “old,” resulting in a high number of both hits and false alarms. Therefore, old-item responses were adjusted to address this bias. First, a “false alarm tendency” value for each participant was computed as their average response to all new items on the 4-point scale; then, a one-sample *t* test was calculated to identify participants whose responses were significantly greater than 2 on the 4-point scale. Because a higher false alarm tendency suggests that these participants more regularly and confidently judged a new item as old, “Probably old” responses were adjusted and considered forgotten in all analyses. After adjustment, participants exhibited an average hit rate (number of adjusted hits/valid trials) of .62, with a standard error of .03. See Table 1 for participant behavioral responses and adjustments.

**Table 1.** Counts of Hit Trials before and after Behavioral Adjustment

*Participant*	*Valid Trials*	*Hits*	*Adjusted Hits*	*Average Response to New Items*	*SE Response to New Items*	*t Value*	*p Value*
1	266	172	172	1.74	0.076	−3.419	.001
**2**	**261**	**166**	**130**	**2.51**	**0.111**	**4.576**	**.000**
3	280	151	151	1.91	0.071	−1.264	.209
**4**	**261**	**231**	**198**	**2.28**	**0.083**	**3.375**	**.001**
**5**	**296**	**228**	**115**	**2.50**	**0.061**	**8.179**	**.000**
**6**	**276**	**165**	**92**	**2.27**	**0.080**	**3.366**	**.001**
7	276	210	210	1.75	0.115	−2.175	.032
**8**	**291**	**281**	**206**	**2.51**	**0.086**	**5.940**	**.000**
9	294	206	206	2.05	0.072	0.698	.487
**10**	**274**	**241**	**196**	**2.30**	**0.107**	**2.808**	**.006**
**11**	**269**	**222**	**207**	**2.33**	**0.116**	**2.835**	**.006**
**12**	**259**	**180**	**96**	**2.41**	**0.064**	**6.435**	**.000**
**13**	**250**	**208**	**158**	**2.66**	**0.132**	**5.001**	**.000**
14	249	188	188	2.10	0.058	1.732	.086
15	234	141	141	1.80	0.115	−1.745	.084
**16**	**279**	**227**	**181**	**2.84**	**0.109**	**7.712**	**.000**
17	257	184	184	1.71	0.113	−2.565	.012
**18**	**276**	**210**	**148**	**2.83**	**0.089**	**9.344**	**.000**
19	248	166	166	2.02	0.134	0.149	.882

The adjustment is for old response bias (in **bold**) based on responses to new items >2 (probably new) at *p* < .05.

### Visual and Semantic Representation Independent of Memory

**Figure 3.**
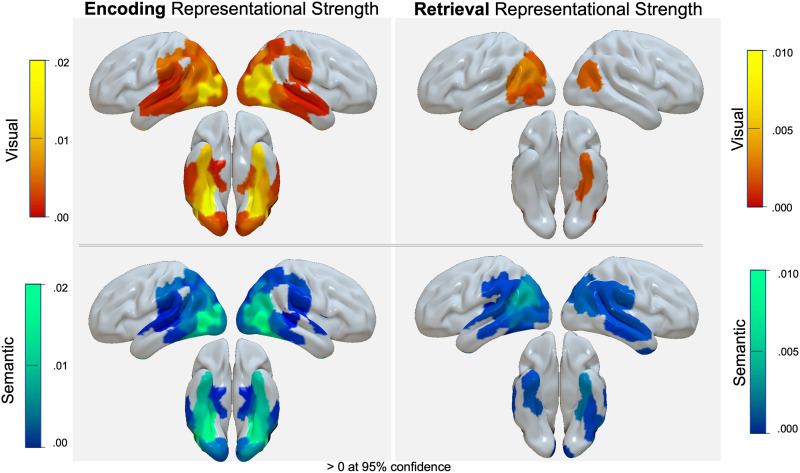
Representational strength indicated by a model estimated IRAF > 0 at 95% confidence. Left. representational strength during the encoding phase for all ROIs along the ventral stream and parietal lobe. Right. Representational strength during the retrieval phase for all ROIs along the ventral stream and parietal lobe. Blue-green. Semantic representation. Red-yellow. Visual representation.

IRAF estimates for visual and semantic representation type during the encoding and retrieval phases—independent of memory performance—were extracted from the larger model using the lmerTest package (Kuznetsova et al., 2017) and are visualized in Figure 3. During encoding, visual and semantic representations (> 0 at 95% confidence, uncorrected for multiple comparisons) exhibit similar cortical representation, whereas during retrieval, semantic representations appear more anterior relative to visual information. In addition, the impact of the memory phase on the cortical location of representations was further examined. The mixed-effects model testing the Memory Phase × Area interaction revealed a significant main effect of Memory Phase (*F* = 233.60, *p* < .001) such that representations are stronger during encoding than retrieval, there is no significant main effect of area, and a significant Memory Phase × ROI Type interaction (*F* = 22.93, *p* < .001) such that the effect of Memory Phase (encoding > retrieval) was greater in the OTC (*t* = 19.216) than that of the IPC (encoding > retrieval, *t* = 6.155; Appendix Figure A1).

Together, Figure 3 and the memory phase interaction reveal that the strongest representation regions during encoding are along the posterior ventral stream, whereas the strongest retrieval representations are in parietal regions, replicating previous RERS findings (Long & Kuhl, 2021; Xiao et al., 2017). The greater involvement of visual cortex during encoding could reflect the fact that encoding stimuli were visually rich objects, whereas retrieval stimuli were lexical cues. To control for this kind of difference between encoding and retrieval stimuli, it is important to focus on memory-related differences by comparing remembered and forgotten items.

### Characteristics of Phase Shifts in Representation

**Figure 4.**
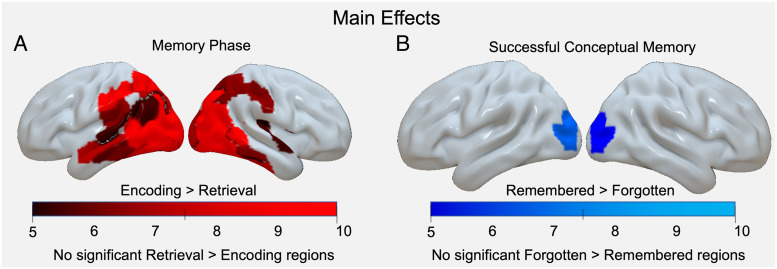
(A) Significant main effects of Memory Phase in which encoding representations are stronger than retrieval. (B) Significant main effects of Memory Success in which representations for remembered items are significantly greater than representations for forgotten items. Color bars = *F* values.

To directly investigate memory phase shifts in representation content and localization, IRAF values were submitted to a larger mixed-effect regression model with random effects of participant and item. The model included the interaction of memory phase (encoding vs. retrieval), representation type (visual vs. semantic), and conceptual memory success (hit vs. miss), as well as their interactions and main effects. The analysis revealed widespread main effects of memory phase between encoding and retrieval (Figure 4A, Table 2). Generally, the strength of representations is greatest during encoding. Intriguingly, there was a cohesive ventral pattern for encoding > retrieval representational strength, with the encoding strength being strongest in the right lateral occipital cortex (*F* = 416.203, *p* < .001), right fusiform gyrus (*F* = 397.582, *p* < .001), left lateral occipital cortex (*F* = 355.236, *p* < .001), and left fusiform gyrus (*F* = 352.644, *p* < .001).

**Table 2.** Regions Exhibiting a Significant Fixed Effect of Memory Phase

*Hemisphere*	*Anatomical Label*	*MNI Coordinates*	*Sum of Squares*	*Denominator df*	*F*	*FDR-adjusted p*
R	Area V5/MT+	48, −70, −1	1.967	19843.776	416.203	.000
R	Medioventral area 37	31, −62, −14	1.822	19843.823	397.582	.000
L	Area V5/MT+	−46, −74, 3	1.713	19844.622	355.236	.000
L	Medioventral area 37	−31, −64, −14	1.572	19845.552	352.644	.000
R	Middle occipital gyrus	34, −86, 11	1.143	19846.381	248.791	.000
R	Lateroventral area 37	43, −49, −19	0.985	19844.490	226.262	.000
L	Middle occipital gyrus	−31, −89, 11	1.027	19842.496	225.701	.000
R	Caudal area 39 (Pgp)	45, −71, 20	0.782	19845.066	165.147	.000
L	Lateroventral area 37	−42, −51, −17	0.654	19843.705	153.458	.000
R	Ventrolateral area 37	54, −57, −8	0.471	19846.909	113.055	.000
L	Inferior occipital gyrus	−30, −88, −12	0.439	19840.964	109.370	.000
R	Inferior occipital gyrus	32, −85, −12	0.353	19844.740	86.736	.000
L	Caudal area 39 (Pgp)	−34, −80, 29	0.359	19849.311	78.125	.000
R	Lateral superior occipital gyrus	29, −75, 36	0.290	19839.991	68.520	.000
R	Caudal lingual gyrus	10, −85, −9	0.255	19850.278	66.826	.000
L	Intraparietal area 7 (Hip3)	−27, −59, 54	0.247	19844.245	59.008	.000
L	Dorsolateral area 37	−59, −58, 4	0.256	19845.615	56.943	.000
L	Occipital polar cortex	−18, −99, 2	0.214	19848.965	54.407	.000
R	Dorsolateral area 37	60, −53, 3	0.220	19849.071	53.121	.000
L	Rostrodorsal area 40 (Pft)	−51, −33, 42	0.215	19848.338	52.622	.000
L	Caudal lingual gyrus	−11, −82, −11	0.199	19853.239	50.686	.000
L	Caudal hippocampus	−28, −30, −10	0.174	19845.861	46.315	.000
L	Extreme lateroventral area 37	−51, −57, −15	0.157	19846.319	39.476	.000
R	Occipital polar cortex	22, −97, 4	0.146	19841.741	36.217	.000
R	Extreme lateroventral area 37	53, −52, −18	0.133	19840.085	33.661	.000
R	Caudal hippocampus	29, −27, −10	0.127	19850.946	33.332	.000
L	Ventrolateral area 37	−55, −60, −6	0.140	19845.537	32.933	.000
R	Intraparietal area 7 (Hip3)	31, −54, 53	0.112	19844.881	28.721	.000
R	Caudolateral area 20	61, −40, −17	0.104	19842.084	27.628	.000
R	Rostrodorsal area 40 (Pft)	47, −35, 45	0.106	19849.142	26.111	.000
R	Caudal area 7	19, −69, 54	0.098	19843.932	25.942	.000
R	Rostrodorsal area 39 (Hip3)	39, −65, 44	0.100	19842.794	24.777	.000
L	Caudal cuneus gyrus	−6, −94, 1	0.086	19842.742	21.836	.000
L	Caudal area 7	−15, −71, 52	0.082	19846.816	20.777	.000
L	Superior temporal sulcus	−52, −50, 11	0.090	19846.546	20.676	.000
L	Lateral superior occipital gyrus	−22, −77, 36	0.068	19849.111	15.842	.000
R	Caudoventral area 20	54, −31, −26	0.056	19847.155	15.074	.001
L	Anterior superior temporal sulcus	−58, −20, −9	0.056	19842.199	14.986	.001
L	Medial area 7 (Pep)	−5, −63, 51	0.058	19847.952	14.863	.001
L	Rostrodorsal area 39 (Hip3)	−38, −61, 46	0.059	19850.419	14.445	.001
R	Caudal cuneus gyrus	8, −90, 12	0.058	19843.200	13.993	.001
R	TE1.0 And TE1.2	51, −4, −1	0.051	19843.464	13.172	.001
L	Rostroventral area 40 (Pfop)	−53, −31, 23	0.047	19837.902	11.676	.003
L	Caudolateral area 20	−59, −42, −16	0.039	19846.996	10.125	.006
L	Rostral hippocampus	−22, −14, −19	0.034	19846.880	8.986	.011
L	Caudal area 21	−65, −30, −12	0.028	19849.383	7.208	.024
L	Rostroventral area 39 (Pga)	−47, −65, 26	0.027	19846.334	6.139	.040
L	Caudal area 22	−62, −33, 7	0.022	19843.779	5.765	.049

Pfop, Pga, Pgp, Pft, and Pep are adopted from von Economo and Koskinas (1925). MT = middle temporal; Hip = hippocampus.

**Figure A2.**
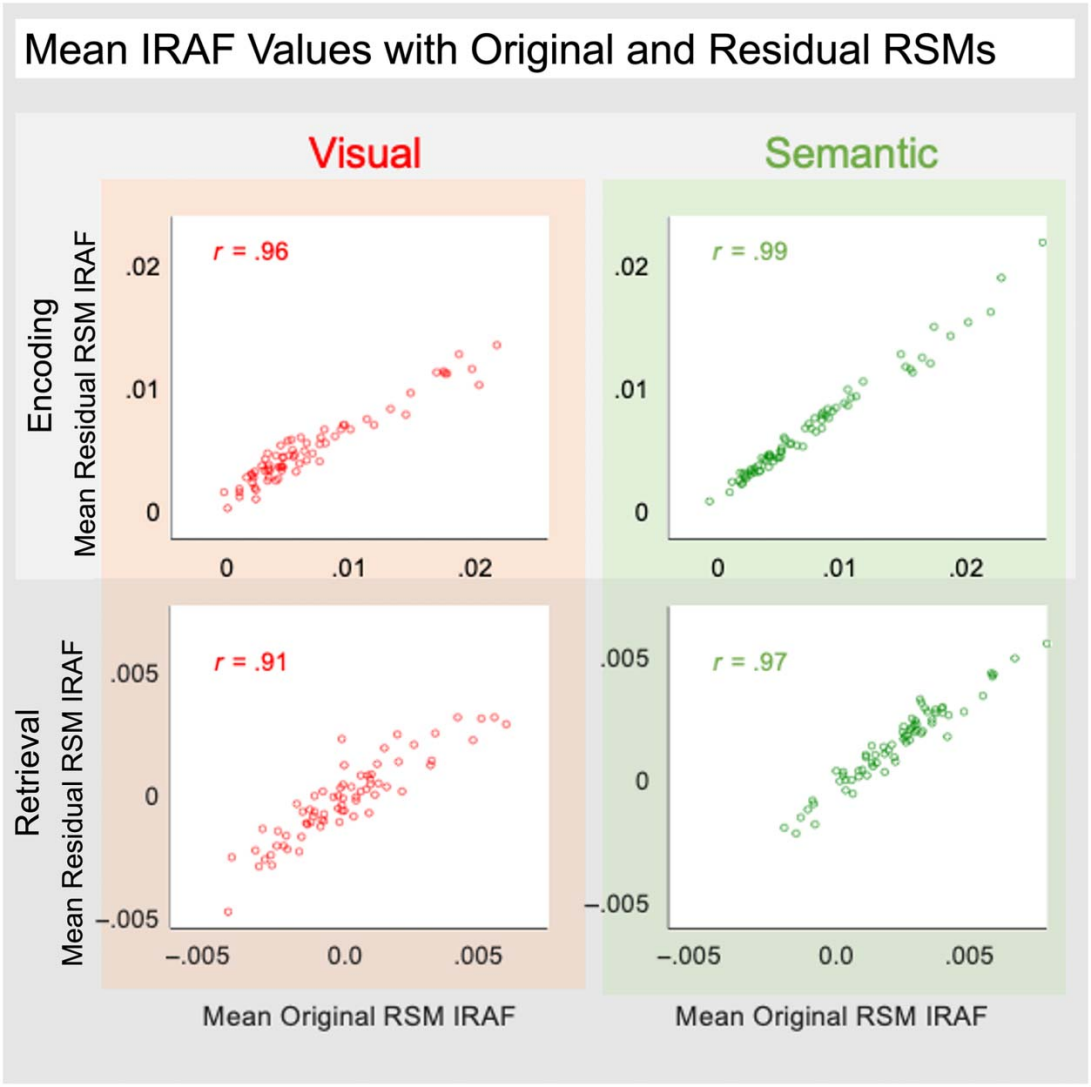
Mean original RSM IRAFs from each ROI plotted against the mean residual RSM IRAFs. Mean IRAFs for encoding data are plotted in the top (light gray background). IRAFs for retrieval data are plotted in the bottom (gray background). Mean IRAFs calculated with visual (red) and semantic (green) RSMs are plotted on the left and right, respectively. The *r* value in each quadrant is the Pearson's correlation of mean IRAFs across ROIs. All quadrants exhibit *r* values > .91.

The inclusion of representation type in the model addressed our first goal of probing if RERS is attributable to changes in content across the memory phases. As mentioned in the Introduction section, one proposed explanation of RERS is that it reflects a change from greater reliance on visual/concrete representation during encoding to greater reliance on more semantic/abstract representations during retrieval (Favila et al., 2020). In contrast with this idea, the representation type factor (visual and semantic information coded from normative visual or taxonomic features, respectively) did not yield any significant main effects or interactions (see Appendix Tables A1, A2, A3, and A4). To ensure that the regional overlap of visual and semantic representations is not driven by the correlation of visual and semantic models (mean item-wise rho = .27), we employed a method to further separate feature types. Specifically, we used MATLAB's fitlm function to create two linear models where each model predicts the outcomes of the other. This process allowed us to isolate the unique visual and semantic information, resulting in residual models. The resulting residual models are anticorrelated (mean rho = −.77). We then applied these residual models to examine how visual and semantic information is processed during the encoding and retrieval, following the same steps outlined in our Methods section (see Figure 2). By averaging IRAFs across participants for each ROI and comparing them with the IRAFs from initial models, we observed a strong and positive correlation (rho range = .91–.99; Appendix Figure A2) between the residual and original data. These high correlations suggest that the coherence observed in the brain's representation areas is primarily driven by the unique neural patterns associated with each type of information rather than by any overlap between the visual and semantic models themselves.

**Figure A3.**
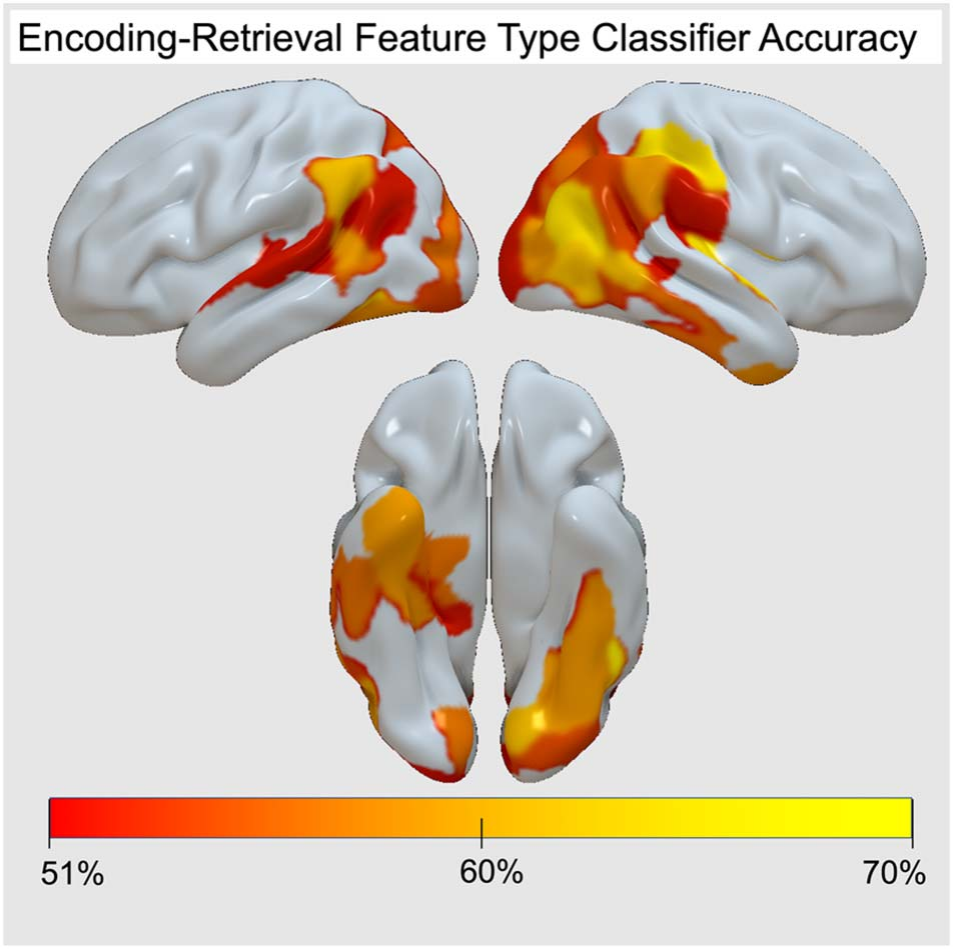
Encoding-trained support vector machine visual and semantic classifier accuracy for retrieval data by ROI. Shown range: 51%–70%. This figure demonstrates that despite a lack of feature-type effects, visual and semantic information are meaningful distinctions in the brain.

Thus, RERS does not seem to reflect a change in the reliance on visual versus semantic representations, and the information underlying encoding and retrieval appears consistent in many regions. To further validate this result, we performed additional analyses to confirm that these feature dimensions are meaningfully represented in the brain and remain consistent in most regions across memory phases. We trained a support vector machine to classify feature types (visual and semantic) of encoding IRAFs in each ROI and then tested it on retrieval ROI data for each participant. The performance of the classifier is calculated by calculating the accuracy of predictions. To confirm the prediction above chance, data from 1000 participants were simulated and feature types were randomly assigned. The sum of the accuracies was then calculated for each simulated and true participant in each ROI; chi-squared tests were then used to determine if the classifier performed above chance. Results show that the classifier trained on encoding IRAFs was able to distinguish visual from semantic IRAF patterns with an average accuracy of 54%, significantly above chance levels, *X*^2^(1) = 16.93, *p* < .0001. Investigating individual ROIs, we found that 42 ROIs exhibited classifier performance above chance (range: 51%–68%). Classifier performance by ROI is visualized in Appendix Figure A3.

The memory success factor identified differences between remembered and forgotten trials regardless of memory phases. The analysis showed representational strength in the left lateral occipital cortex (*F* = 7.488, *p* = .022), left medio-ventral occipital cortex (*F* = 6.533, *p* = .034), and right lateral occipital cortex (*F* = 6.318, *p* = .037), positively predicted memory success, irrespective of memory phase (Figure 4B, and Table 3). This result reinforces the role that secondary visual cortices play in both the successful *encoding* of visual and semantic information when object images were either successfully remembered or forgotten, as well as the successful *retrieval* of the same information, in which verbal cues elicited successful hit- or miss-related representations.

**Table 3.** Regions Exhibiting a Significant Fixed Effect of Memory Success

*Hemisphere*	*Anatomical Label*	*MNI Coordinates*	*Sum of Squares*	*Denominator df*	*F*	*FDR-adjusted p*
L	Middle occipital gyrus	−31, −89, 11	0.034	11910.532	7.488	.022
L	Caudal cuneus gyrus	−6, −94, 1	0.026	12938.037	6.533	.034
R	Middle occipital gyrus	34, −86, 11	0.029	16649.236	6.318	.037

**Figure 5.**
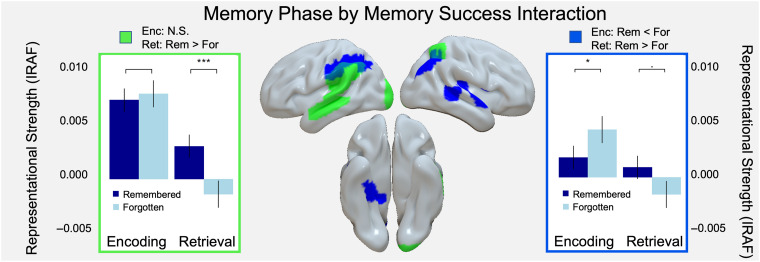
Significant interactions of Memory Phase and Memory Success. Middle: Regions exhibiting significant interactions for two distinct patterns visualized in green and blue boxes. Left: Interaction visualization for success effects at retrieval and no success effect at encoding (green). Right: Interaction visualization for success effects at retrieval, coupled with greater representational strength for forgotten than remembered trials during encoding (blue).

Finally, to investigate our second goal of examining the link between RERS and memory performance, we turned to the Memory Phase × Memory Success interaction. We found significant interactions in 14 regions within both the dorsal and ventral stream (Figure 5, Table 4). To understand the nature of the interactions with respect to memory success, post hoc tests (*difflsmeans* in lmerTest; Kuznetsova et al., 2017) were performed comparing model estimates for remembered and forgotten trials during encoding and retrieval (Table 5). The magnitude of these *t* values was used to sort ROIs into subgroups, demonstrating qualitatively similar patterns of interactions. These post hoc tests revealed two distinct interaction patterns. The first interaction pattern (Figure 5, green color) corresponds to memory success effects that occur during retrieval but not during encoding. These interactions demonstrate that representations do shift location to parietal/frontal regions during retrieval and that this shift supports successful memory. The second interaction pattern (Figure 5, blue color) shows the same memory success effects during retrieval, but the interaction is at least partially driven by greater encoding representations during subsequently forgotten than subsequently remembered trials. These interactions suggest that in some regions, certain representations can be beneficial to retrieval but detrimental during encoding. This pattern resembles the encoding–retrieval flip reliably found in univariate activity (Huijbers et al., 2012; Daselaar et al., 2009), indicating a connection between RERS and the univariate encoding–retrieval flip. Taken together, our results suggest that representational shifts in cortical location, but not content, support successful conceptual memory.

**Table 4.** Regions Exhibiting a Significant Memory Phase × Memory Success Interaction

*Hemisphere*	*Anatomical Label*	*MNI Coordinates*	*Sum of Squares*	*Denominator df*	*F*	*FDR-adjusted p*
L	Caudal area 40 (Pfm)	−56, −49, 38	0.047	19848.992	11.763	.003
L	Area 41/42	−54, −32, 12	0.046	19840.751	11.511	.003
R	TE1.0 And TE1.2	51, −4, −1	0.039	19843.464	10.045	.006
R	Medial area 7 (Pep)	6, −65, 51	0.036	19853.577	9.412	.009
R	Rostrodorsal area 39 (Hip3)	39, −65, 44	0.034	19842.794	8.463	.014
R	Intraparietal area 7 (Hip3)	31, −54, 53	0.032	19844.881	8.312	.015
L	Anterior superior temporal sulcus	−58, −20, −9	0.031	19842.199	8.297	.015
L	Rostroventral area 40 (Pfop)	−53, −31, 23	0.030	19837.902	7.507	.022
L	Occipital polar cortex	−18, −99, 2	0.029	19848.965	7.411	.023
R	Superior temporal sulcus	53, −37, 3	0.029	19843.050	7.382	.023
R	Caudal hippocampus	29, −27, −10	0.027	19850.946	7.105	.025
L	Rostrodorsal area 39 (Hip3)	−38, −61, 46	0.029	19850.419	6.978	.027
L	Rostrodorsal area 40 (Pft)	−51, −33, 42	0.026	19848.338	6.322	.037
R	Caudal Area 40 (Pfm)	57, −44, 38	0.022	19847.018	5.728	.049

Pfop, Pfm, Pft, and Pep are adopted from von Economo and Koskinas (1925). Hip = hippocampus.

**Table 5.** Post Hoc Tests for Regions Exhibiting a Significant Memory Phase × Memory Success Interaction

*Hemi*	*Anatomical Label*	*MNI Co.*	*Enc Rem-For*		*Ret Rem-For*	
			*t*	*p*	*t*	*p*
	*Pattern 1: Retrieval Success*					
R	TE1.0 And TE1.2	51, −4, −1	−1.647	.100	2.732	.006
R	Rostrodorsal area 39 (Hip3)	39, −65, 44	−1.648	.099	2.352	.019
R	Medial area 7 (Pep)	6, −65, 51	−1.911	.056	2.321	.020
R	Superior temporal sulcus	53, −37, 3	−1.560	.119	2.199	.028
L	Anterior superior temporal sulcus	−58, −20, −9	−2.024	.043	1.944	.052
L	Rostrodorsal area 40 (Pft)	−51, −33, 42	−1.515	.130	1.937	.053
L	Rostrodorsal area 39 (Hip3)	−38, −61, 46	−1.871	.061	1.765	.078
R	Caudal hippocampus	29, −27, −10	−1.980	.048	1.684	.092

	*Pattern 2: Encoding/Retrieval Flip*					
L	Caudal area 40 (Pfm)	−56, −49, 38	−1.022	.307	3.695	.000
R	Intraparietal area 7 (Hip3)	31, −54, 53	−0.322	.747	3.657	.000
L	Area 41/42	−54, −32, 12	−1.264	.206	3.430	.001
L	Occipital polar cortex	−18, −99, 2	−0.440	.660	3.309	.001
L	Rostroventral area 40 (Pfop)	−53, −31, 23	−0.712	.477	3.056	.002
R	Caudal area 40 (Pfm)	57, −44, 38	−0.775	.439	2.532	.011

Two patterns reported: (1) success effects at retrieval and (2) success effects at retrieval, coupled with greater representational strength for forgotten trials than remembered trials during encoding contributing to the interaction (i.e., an “encoding/retrieval flip”). These correspond to green and blue colors in Figure 5, respectively. Pfop, Pfm, Pft, TE1, and Pep are adopted from von Economo and Koskinas (1925). Hip = hippocampus.

## DISCUSSION

Prior knowledge about objects in the world has profound effects upon how we encode and retrieve those objects, leading us to organize our episodic experiences along semantic dimensions. The current study addresses how this mnemonic organization shifts across encoding and retrieval phases. We examined memory effects in these regions with a 3-way Memory Phase (encoding vs. retrieval) × Memory Success (remembered vs. forgotten) × Representation Type (visual vs. semantic) ANOVA. This analysis revealed the two principal findings. First, we found no main effects or interactions with representation type (based on visual and semantic information), perhaps challenging the idea that RERS can be explained by a change in the nature of representations from visual/concrete during encoding to semantic/abstract during retrieval (Favila et al., 2020). Second, we found significant interactions between memory phase and memory success, supporting the idea that RERS is driven by memory operations rather than by differences in other cognitive processes that may differ between encoding and retrieval (e.g., memory search, control operations). Critically, lateral parietal cortex demonstrated a Phase × Memory interaction, such that memory representations contributed to memory success during retrieval but not during encoding. In a subset of parietal areas, the encoding–retrieval dissociation was more dramatic because stronger encoding representations were associated with subsequent forgetting, resembling the “encoding–retrieval flip” previously found in univariate activation studies (Huijbers et al., 2012; Daselaar et al., 2009). We discuss our two main findings below.

### Information Supporting Encoding and Retrieval

Consistent with a handful of previous studies focused on the item representations during retrieval (Long & Kuhl, 2021; Favila et al., 2018; Xiao et al., 2017), we found that although encoding representations were strongest in posterior occipito-temporal regions, the strongest representations at retrieval were in anterior parietal regions (i.e., the RERS). One explanation proposed for RERS is that representations are more visual/concrete during encoding but more semantic/abstract during retrieval (Favila et al., 2020). Our results do not support this hypothesis. Independent of memory performance, the item-wise correspondence between the neural pattern similarity and visual model information (e.g., “is red”) during encoding was characterized by the distribution of cortical representation centered on occipito-temporal and parietal cortex. Item-wise correspondence for semantic information (e.g., “is found in forests”) largely overlapped with this pattern, albeit with a slightly more anterior distribution during retrieval (see Figure 3). As such, our first main finding from our central three-way (Memory Phase × Memory Success × Representation Type) interaction was that RERS occurred for both visual and semantic information, challenging the hypothesis of a shift in content. Notably, this null effect was observed for all regions, including regions that demonstrated evidence that they represent either visual or semantic information (Figure 3), independent of memory success. Furthermore, the strong correlations between IRAFs derived from the original visual and semantic RSMs and those derived from the residual RSMs confirm that the coherence observed in the brain's representation areas is mainly driven by the distinct neural patterns associated with each type of information, rather than any overlap between the visual and semantic models themselves. Thus, we found no shift in the balance of visual and semantic representations, which is quite striking given that encoding stimuli were object pictures (favoring visual representations), and retrieval cues were lexical (favoring abstract semantic representations).

Taken in this context, a null effect of Representation Type—or interactions with this factor—suggests that the information underlying both encoding and retrieval representations in most cortical regions is highly consistent across memory phases.

The inference that the information underlying encoding and retrieval representations is consistent across memory phase is also qualitatively supported by the similarity in representational strength depicted in Figure 3 and quantitatively supported by the encoding-trained visual and semantic classifier performance when tested on retrieval data (Appendix Figure A3). The role of occipito-temporal cortex and parietal regions in memory storage is a topic often investigated in episodic memory studies, but also in the working memory fMRI literature (Sreenivasan & D'Esposito, 2019; Ester, Sprague, & Serences, 2015). Some evidence from this domain suggests that parietal regions store categorical rather than visual properties (Sarma, Masse, Wang, & Freedman, 2016). This evidence suggests that the involvement of parietal regions during retrieval that we found should have been associated with a greater contribution of semantic representations, but this is not what we found. Remarkably, although the retrieval cue was a word without any visual detail, these regions represented visual and semantic information to the same extent.

This null finding also supports the growing appreciation for the role of lateral parietal cortex in semantic cognition. Although the conclusions that can be made of such a null finding are necessarily limited, these findings nonetheless contribute to the paucity of RSA analyses focused explicitly on the informational content of retrieval representations (as opposed to the similarity between encoding and retrieval representations, i.e., ERS). The more explicit finding that both encoding and retrieval representations rely on visual and abstract semantic information contributes to the consensus that lateral parietal cortex traffics in multiple forms of meaningful representations. Although attention for such an amodal semantic hub has traditionally focused on the anterior temporal lobe, meta-analytic approaches identify lateral parietal cortex—including the angular gyrus—as a dense concentration of activation foci for semantic contrasts (Binder, Desai, Graves, & Conant, 2009). Moreover, the representational role of lateral parietal cortices in memory retrieval is not dependent on the visual detail of the retrieval cue. Recently, researchers employed inverted semantic encoding models with fMRI data to reconstruct multidimensional content in natural scene images during both memory recognition and memory recall. They discovered that visual and lateral parietal cortices played a role in successful reconstructions, with lateral parietal activity being less affected by the distinction between viewing and recalling images compared with visual cortical activity (Wang, Lee, & Kuhl, 2023). Furthermore, this region is commonly activated in studies manipulating semantic control (Badre & Wagner, 2002), and stimulation of this region enhances semantic integration (Price, Peelle, Bonner, Grossman, & Hamilton, 2016). The strongest evidence for this region in representing amodal information comes from studies that explicitly compare pictorial and lexical stimuli and find modality-invariant semantic representations in the lateral parietal cortex utilizing a representational similarity approach (Devereux, Clarke, Marouchos, & Tyler, 2013).

### Impact of RERSs on Conceptual Memory Success

Our second main finding was that RERS was associated with memory success. In particular, retrieval representations in lateral parietal cortex were stronger for remembered than forgotten items. During encoding, representations in lateral parietal regions either did not contribute to subsequent memory or were associated with subsequent forgetting. In other words, in some parietal regions, representations were associated with encoding failure but with retrieval success. This “flip” in the value of representational strength mirrors that of univariate patterns found in ventral parietal regions (so-called *encoding–retrieval flip* regions), in which the angular gyrus is more activated during encoding of subsequently forgotten trials, but more activated during retrieval during remember trials (Huijbers et al., 2012; Daselaar et al., 2009; Cabeza, Ciaramelli, Olson, & Moscovitch, 2008). Evidence for such phase-dependent representations at retrieval supports the view that the cortical location of content representations is fundamentally determined by whether attention is internally oriented to memories or externally oriented to current perceptual experience. This lends support to interpretations of recent literature suggesting the inferior parietal cortex has an internal representational role during memory retrieval, even when the perceptual presentation is identical during both memory states (Long & Kuhl, 2021; Zhao, Chanales, & Kuhl, 2021; Tarder-Stoll, Jayakumar, Dimsdale-Zucker, Günseli, & Aly, 2020). Indeed, regions associated with enhanced mnemonic representation at retrieval have been linked to directed internal attention during memory retrieval, self-awareness during movement planning and execution, and off-task periods (e.g., resting state; Honey, Newman, & Schapiro, 2017). Moreover, converging evidence from fMRI and lesion studies shows that IPC delay activity is associated with attention directed to internal memory representations during verbal working memory tasks (Berryhill, Chein, & Olson, 2011; Chein, Ravizza, & Fiez, 2003). In addition, we focus our discussion on encoding/retrieval flips in parietal regions as those were hypothesized considering previous work (Huijbers et al., 2012; Daselaar et al., 2009). However, it should be noted that some occipito-temporal regions exhibit the pattern and future research should probe their roles in representation and internal attention.

Influential models of object representation consider the inferior parietal cortex an amodal semantic hub (Humphreys, Jung, & Lambon Ralph, 2022; Binder et al., 2009) and suggest that perceived content shifts during memory retrieval may arise via the underlying network interactions that elude representational approaches, similar to other semantic hubs like the anterior temporal lobe (Patterson & Ralph, 2016; Patterson, Nestor, & Rogers, 2007). Critically, recent evidence suggests that parietal regions, particularly the angular gyrus, additively encode both semantic content and episodic memory information (Lee, Keene, Sweigart, Hutchinson, & Kuhl, 2023). Future work focused on such network analysis (e.g., informational connectivity, Coutanche & Thompson-Schill, 2013) may help to resolve how mnemonic information shifts along a cortical axis and what factors affect that shift.

It is notable that the current study did not find any areas where outward-directed attention enhances memory creation but impairs retrieval. Because retrieving memories requires a mix of external and internal attention at varying time points (first external attention to the cue, and subsequent internal attention for the memory), fMRI's limited time sensitivity might obscure such findings. We anticipate that approaches or designs (Cooper & Ritchey, 2020) with improved temporal precision and expanded ROIs that include the entire visual attention network will be more effective in pinpointing these specific areas.

### Methodological Considerations

Lastly, we believe that a few methodological choices in our analysis have some advantages over more typical multivoxel pattern analyses in memory studies. As noted in the Introduction section, much of our inference on what representations are relevant during retrieval comes through the lens of encoding, often through the use of ERS analysis. In such a framework, however, it is likely that retrieval operations are not simply a reinstatement of cognitive processes at encoding and a more expansive approach utilized in RERS studies (Long & Kuhl, 2021; Favila et al., 2018; Xiao et al., 2017) is necessary. Furthermore, as we were primarily interested in encoding–retrieval shifts, our results are presented as a function of a full ANOVA that includes both encoding and retrieval information. Therefore, we did not test the traditional subsequent memory effects (i.e., tests only encoding success). Subsequent memory effects for representational analyses in this study have been previously reported (Davis et al., 2021). Second, we chose to utilize a widespread temporal and parietal network to investigate potential regions sensitive to phase shifts outside of typical inferior parietal regions. Constrained ROI approaches using a small number of ROIs (5–12) help to address region-specific hypotheses and avoid multiple comparison issues but limit the theoretical inferences made about what regions contribute to successful memory (especially when such regions are limited by encoding success). Still, effects of interest may be present outside our chosen temporal and parietal regions, and future studies may want to probe representations in frontal cortices. Finally, our item-wise approach to representational similarity analyses (Davis et al., 2021) allows for greater statistical inference, especially in the context of behavioral predictors. Unlike traditional approaches, mixed-effect regressions utilizing subject-level random effects allow for within and between subject-level variability, especially in the face of varying observation counts (e.g., number of remembered and forgotten trials [Baayen, Davidson, & Bates, 2008]).

### Conclusion

In conclusion, our item-wise approach to RSA during encoding and retrieval revealed that representational shifts in cortical location (from occipito-temporal cortex at encoding to lateral parietal cortex at retrieval), but not shifts in content, support successful memory. Second, representations in parietal regions were more robust for subsequently forgotten trials during encoding than for remembered trials during retrieval, indicating an encoding–retrieval flip and suggesting that mnemonic representations interact with and are dependent on internal attention processes. Together, these findings suggest that cortical representations that shift location with changes in memory phase support successful conceptual memory.

## APPENDIX

**Table A1.** Main Effect of Feature Type Null Results

*Hemisphere*	*Anatomical Label*	*MNI Coordinates*	*Sum of Squares*	*Denominator df*	*F*	*FDR-adjusted p*
L	Area 41/42	−54, −32, 12	0.01	20010.518	1.461	.713
L	TE1.0 and TE1.2	−50, −11, 1	0.00	20006.260	0.692	.815
R	TE1.0 and TE1.2	51, −4, −1	0.00	20005.488	0.466	.838
L	Caudal area 22	−62, −33, 7	0.00	20008.954	1.202	.738
R	Caudal area 22	66, −20, 6	0.01	20017.519	3.150	.463
L	Caudal area 21	−65, −30, −12	0.00	20009.460	0.847	.793
R	Caudal area 21	65, −29, −13	0.01	20016.145	1.577	.697
L	Dorsolateral area 37	−59, −58, 4	0.00	20002.126	0.636	.838
R	Dorsolateral area 37	60, −53, 3	0.00	20009.699	0.132	.967
L	Anterior superior temporal sulcus	−58, −20, −9	0.00	20007.641	0.198	.943
R	Anterior superior temporal sulcus	58, −16, −10	0.00	20001.721	1.284	.738
L	Extreme lateroventral area 37	−51, −57, −15	0.01	20006.205	1.716	.693
R	Extreme lateroventral area 37	53, −52, −18	0.01	20004.037	1.681	.696
L	Ventrolateral area 37	−55, −60, −6	0.01	19998.286	3.210	.453
R	Ventrolateral area 37	54, −57, −8	0.00	20008.756	0.695	.815
L	Caudolateral area 20	−59, −42, −16	0.00	20010.898	0.781	.799
R	Caudolateral area 20	61, −40, −17	0.01	20008.997	1.391	.717
L	Caudoventral area 20	−55, −31, −27	0.00	20008.728	0.680	.815
R	Caudoventral area 20	54, −31, −26	0.01	20005.576	1.948	.625
L	Medioventral area 37	−31, −64, −14	0.01	19990.011	2.317	.567
R	Medioventral area 37	31, −62, −14	0.00	19976.640	0.349	.875
L	Lateroventral area 37	−42, −51, −17	0.02	19991.726	5.708	.235
R	Lateroventral area 37	43, −49, −19	0.02	19984.640	4.219	.318
L	Rostroposterior superior temporal sulcus	−54, −40, 4	0.00	20003.155	0.001	.999
R	Rostroposterior superior temporal sulcus	53, −37, 3	0.00	20009.510	0.881	.793
L	Caudoposterior superior temporal sulcus	−52, −50, 11	0.00	20002.560	0.261	.914
R	Caudoposterior superior temporal sulcus	57, −40, 12	0.00	20018.674	0.628	.838
L	Caudal area 7	−15, −71, 52	0.00	20004.520	0.344	.875
R	Caudal area 7	19, −69, 54	0.03	20000.380	7.595	.143
L	Intraparietal area 7 (hIP3)	−27, −59, 54	0.00	20008.985	0.628	.838
R	Intraparietal area 7 (hIP3)	31, −54, 53	0.00	20005.553	1.141	.738
L	Caudal area 39 (PGp)	−34, −80, 29	0.01	19990.959	2.121	.580
R	Caudal area 39 (PGp)	45, −71, 20	0.00	19985.354	0.026	.974
L	Rostrodorsal area 39 (Hip3)	−38, −61, 46	0.00	20008.282	0.005	.998
R	Rostrodorsal area 39 (Hip3)	39, −65, 44	0.01	20004.266	1.366	.717
L	Rostrodorsal area 40 (PFt)	−51, −33, 42	0.00	20003.759	0.449	.838
R	Rostrodorsal area 40 (PFt)	47, −35, 45	0.00	20004.898	0.025	.974
L	Caudal area 40 (PFm)	−56, −49, 38	0.01	20004.264	1.383	.717
R	Caudal area 40 (PFm)	57, −44, 38	0.00	20013.796	0.086	.967
L	Rostroventral area 39 (PGa)	−47, −65, 26	0.01	19996.743	1.538	.705
R	Rostroventral area 39 (PGa)	53, −54, 25	0.01	20011.915	3.369	.425
L	Rostroventral area 40 (PFop)	−53, −31, 23	0.00	20002.764	0.001	.999
R	Rostroventral area 40 (PFop)	55, −26, 26	0.00	20013.883	0.267	.911
L	Medial area 7 (PEp)	−5, −63, 51	0.02	20014.191	5.288	.249
R	Medial area 7 (PEp)	6, −65, 51	0.02	20015.464	4.807	.263
L	Caudal lingual gyrus	−11, −82, −11	0.02	20007.266	5.346	.249
R	Caudal lingual gyrus	10, −85, −9	0.02	20014.005	3.986	.357
L	Caudal cuneus gyrus	−6, −94, 1	0.03	19992.867	6.699	.179
R	Caudal cuneus gyrus	8, −90, 12	0.03	20005.716	6.144	.206
L	Middle occipital gyrus	−31, −89, 11	0.01	19986.693	2.116	.580
R	Middle occipital gyrus	34, −86, 11	0.00	19990.992	0.087	.967
L	Area V5/MT+	−46, −74, 3	0.06	19968.917	13.118	.072
R	Area V5/MT+	48, −70, −1	0.02	19971.512	4.910	.259
L	Occipital polar cortex	−18, −99, 2	0.04	19999.860	9.656	.120
R	Occipital polar cortex	22, −97, 4	0.00	19994.577	0.000	.999
L	Inferior occipital gyrus	−30, −88, −12	0.05	19992.691	11.400	.072
R	Inferior occipital gyrus	32, −85, −12	0.01	20012.207	1.795	.670
L	Medial superior occipital gyrus	−11, −88, 31	0.01	20006.692	2.506	.540
R	Medial superior occipital gyrus	16, −85, 34	0.00	20009.250	0.810	.793
L	Lateral superior occipital gyrus	−22, −77, 36	0.01	19994.613	1.942	.625
R	Lateral superior occipital gyrus	29, −75, 36	0.02	20005.163	4.928	.259
L	Rostral hippocampus	−22, −14, −19	0.00	20013.983	0.069	.967
R	Rostral hippocampus	22, −12, −20	0.00	20008.247	0.492	.838
L	Caudal hippocampus	−28, −30, −10	0.00	20011.471	0.328	.881

**Table A2.** Memory Success × Feature Type Null Results

*Hemisphere*	*Anatomical Label*	*MNI Coordinates*	*Sum of Squares*	*Denominator df*	*F*	*FDR-adjusted p*
L	Area 41/42	−54, −32, 12	0.00	19857.821	0.053	.967
L	TE1.0 and TE1.2	−50, −11, 1	0.00	19859.547	0.307	.893
R	TE1.0 and TE1.2	51, −4, −1	0.01	19859.656	2.347	.567
L	Caudal area 22	−62, −33, 7	0.00	19860.287	0.004	.998
R	Caudal area 22	66, −20, 6	0.00	19866.526	0.037	.967
L	Caudal area 21	−65, −30, −12	0.00	19865.352	0.287	.898
R	Caudal area 21	65, −29, −13	0.00	19867.277	0.054	.967
L	Dorsolateral area 37	−59, −58, 4	0.02	19860.972	4.874	.259
R	Dorsolateral area 37	60, −53, 3	0.00	19865.002	0.438	.838
L	Anterior superior temporal sulcus	−58, −20, −9	0.01	19858.692	3.855	.365
R	Anterior superior temporal sulcus	58, −16, −10	0.00	19859.077	0.486	.838
L	Extreme lateroventral area 37	−51, −57, −15	0.01	19862.103	2.047	.595
R	Extreme lateroventral area 37	53, −52, −18	0.00	19856.358	0.000	.999
L	Ventrolateral area 37	−55, −60, −6	0.01	19860.506	1.288	.738
R	Ventrolateral area 37	54, −57, −8	0.00	19862.914	0.469	.838
L	Caudolateral area 20	−59, −42, −16	0.00	19863.400	0.301	.895
R	Caudolateral area 20	61, −40, −17	0.00	19858.762	0.042	.967
L	Caudoventral area 20	−55, −31, −27	0.00	19862.521	0.307	.893
R	Caudoventral area 20	54, −31, −26	0.01	19862.835	2.836	.489
L	Medioventral area 37	−31, −64, −14	0.01	19859.558	1.152	.738
R	Medioventral area 37	31, −62, −14	0.00	19856.486	0.388	.863
L	Lateroventral area 37	−42, −51, −17	0.01	19858.055	2.151	.579
R	Lateroventral area 37	43, −49, −19	0.00	19857.956	0.173	.946
L	Rostroposterior superior temporal sulcus	−54, −40, 4	0.01	19861.449	1.724	.693
R	Rostroposterior superior temporal sulcus	53, −37, 3	0.00	19859.788	0.124	.967
L	Caudoposterior superior temporal sulcus	−52, −50, 11	0.00	19861.881	0.010	.998
R	Caudoposterior superior temporal sulcus	57, −40, 12	0.01	19868.765	3.504	.412
L	Caudal area 7	−15, −71, 52	0.00	19862.424	0.942	.784
R	Caudal area 7	19, −69, 54	0.01	19859.342	1.609	.697
L	Intraparietal area 7 (hIP3)	−27, −59, 54	0.00	19860.707	0.198	.943
R	Intraparietal area 7 (hIP3)	31, −54, 53	0.00	19860.918	0.041	.967
L	Caudal area 39 (PGp)	−34, −80, 29	0.00	19863.043	0.059	.967
R	Caudal area 39 (PGp)	45, −71, 20	0.00	19858.554	0.032	.972
L	Rostrodorsal area 39 (Hip3)	−38, −61, 46	0.00	19866.114	0.145	.963
R	Rostrodorsal area 39 (Hip3)	39, −65, 44	0.00	19858.813	0.292	.898
L	Rostrodorsal area 40 (PFt)	−51, −33, 42	0.00	19863.694	0.682	.815
R	Rostrodorsal area 40 (PFt)	47, −35, 45	0.00	19864.516	0.036	.967
L	Caudal area 40 (PFm)	−56, −49, 38	0.00	19864.372	0.031	.972
R	Caudal area 40 (PFm)	57, −44, 38	0.00	19863.780	0.230	.922
L	Rostroventral area 39 (PGa)	−47, −65, 26	0.00	19861.019	0.503	.838
R	Rostroventral area 39 (PGa)	53, −54, 25	0.00	19866.542	0.093	.967
L	Rostroventral area 40 (PFop)	−53, −31, 23	0.00	19854.258	0.042	.967
R	Rostroventral area 40 (PFop)	55, −26, 26	0.00	19864.251	0.342	.875
L	Medial area 7 (PEp)	−5, −63, 51	0.00	19864.613	0.008	.998
R	Medial area 7 (PEp)	6, −65, 51	0.00	19869.795	0.019	.983
L	Caudal lingual gyrus	−11, −82, −11	0.00	19868.530	0.177	.946
R	Caudal lingual gyrus	10, −85, −9	0.00	19866.618	0.144	.963
L	Caudal cuneus gyrus	−6, −94, 1	0.00	19857.388	0.001	.999
R	Caudal cuneus gyrus	8, −90, 12	0.00	19859.310	0.037	.967
L	Middle occipital gyrus	−31, −89, 11	0.00	19856.487	0.008	.998
R	Middle occipital gyrus	34, −86, 11	0.00	19860.373	0.803	.793
L	Area V5/MT+	−46, −74, 3	0.00	19856.327	0.502	.838
R	Area V5/MT+	48, −70, −1	0.00	19855.868	0.145	.963
L	Occipital polar cortex	−18, −99, 2	0.00	19863.893	0.761	.803
R	Occipital polar cortex	22, −97, 4	0.00	19856.728	0.598	.838
L	Inferior occipital gyrus	−30, −88, −12	0.00	19855.787	0.013	.995
R	Inferior occipital gyrus	32, −85, −12	0.00	19861.416	0.054	.967
L	Medial superior occipital gyrus	−11, −88, 31	0.00	19865.123	0.995	.774
R	Medial superior occipital gyrus	16, −85, 34	0.00	19861.708	0.554	.838
L	Lateral superior occipital gyrus	−22, −77, 36	0.00	19863.300	0.038	.967
R	Lateral superior occipital gyrus	29, −75, 36	0.00	19856.341	0.007	.998
L	Rostral hippocampus	−22, −14, −19	0.00	19863.630	1.130	.738
R	Rostral hippocampus	22, −12, −20	0.01	19863.302	1.483	.711
L	Caudal hippocampus	−28, −30, −10	0.00	19862.317	0.246	.918

**Table A3.** Memory Phase × Feature Type Null Results

*Hemisphere*	*Anatomical Label*	*MNI Coordinates*	*Sum of Squares*	*Denominator df*	*F*	*FDR-adjusted p*
L	Area 41/42	−54, −32, 12	0.00	19840.751	0.996	.774
L	TE1.0 and TE1.2	−50, −11, 1	0.00	19843.317	0.537	.838
R	TE1.0 and TE1.2	51, −4, −1	0.01	19843.464	2.744	.494
L	Caudal area 22	−62, −33, 7	0.01	19843.779	1.892	.636
R	Caudal area 22	66, −20, 6	0.00	19849.570	1.199	.738
L	Caudal area 21	−65, −30, −12	0.03	19849.383	7.474	.143
R	Caudal area 21	65, −29, −13	0.00	19850.720	0.895	.793
L	Dorsolateral area 37	−59, −58, 4	0.00	19845.615	0.038	.967
R	Dorsolateral area 37	60, −53, 3	0.00	19849.071	0.000	.999
L	Anterior superior temporal sulcus	−58, −20, −9	0.02	19842.199	4.954	.259
R	Anterior superior temporal sulcus	58, −16, −10	0.01	19843.447	2.240	.567
L	Extreme lateroventral area 37	−51, −57, −15	0.00	19846.319	0.027	.974
R	Extreme lateroventral area 37	53, −52, −18	0.00	19840.085	0.003	.998
L	Ventrolateral area 37	−55, −60, −6	0.00	19845.537	0.813	.793
R	Ventrolateral area 37	54, −57, −8	0.00	19846.909	0.858	.793
L	Caudolateral area 20	−59, −42, −16	0.00	19846.996	0.713	.809
R	Caudolateral area 20	61, −40, −17	0.00	19842.084	0.433	.838
L	Caudoventral area 20	−55, −31, −27	0.00	19846.206	0.000	.999
R	Caudoventral area 20	54, −31, −26	0.02	19847.155	5.251	.249
L	Medioventral area 37	−31, −64, −14	0.00	19845.552	0.054	.967
R	Medioventral area 37	31, −62, −14	0.00	19843.823	0.368	.874
L	Lateroventral area 37	−42, −51, −17	0.00	19843.705	0.807	.793
R	Lateroventral area 37	43, −49, −19	0.00	19844.490	0.462	.838
L	Rostroposterior superior temporal sulcus	−54, −40, 4	0.01	19845.896	1.625	.697
R	Rostroposterior superior temporal sulcus	53, −37, 3	0.01	19843.050	3.019	.463
L	Caudoposterior superior temporal sulcus	−52, −50, 11	0.00	19846.546	0.447	.838
R	Caudoposterior superior temporal sulcus	57, −40, 12	0.01	19852.056	2.214	.567
L	Caudal area 7	−15, −71, 52	0.00	19846.816	0.184	.946
R	Caudal area 7	19, −69, 54	0.00	19843.932	0.176	.946
L	Intraparietal area 7 (hIP3)	−27, −59, 54	0.00	19844.245	0.115	.967
R	Intraparietal area 7 (hIP3)	31, −54, 53	0.00	19844.881	0.004	.998
L	Caudal area 39 (PGp)	−34, −80, 29	0.00	19849.311	0.116	.967
R	Caudal area 39 (PGp)	45, −71, 20	0.00	19845.066	0.000	.999
L	Rostrodorsal area 39 (Hip3)	−38, −61, 46	0.00	19850.419	0.769	.802
R	Rostrodorsal area 39 (Hip3)	39, −65, 44	0.00	19842.794	0.483	.838
L	Rostrodorsal area 40 (PFt)	−51, −33, 42	0.00	19848.338	1.139	.738
R	Rostrodorsal area 40 (PFt)	47, −35, 45	0.00	19849.142	0.523	.838
L	Caudal area 40 (PFm)	−56, −49, 38	0.00	19848.992	0.804	.793
R	Caudal area 40 (PFm)	57, −44, 38	0.00	19847.018	0.884	.793
L	Rostroventral area 39 (PGa)	−47, −65, 26	0.00	19846.334	0.490	.838
R	Rostroventral area 39 (PGa)	53, −54, 25	0.01	19850.448	1.536	.705
L	Rostroventral area 40 (PFop)	−53, −31, 23	0.00	19837.902	0.395	.863
R	Rostroventral area 40 (PFop)	55, −26, 26	0.00	19847.554	1.190	.738
L	Medial area 7 (PEp)	−5, −63, 51	0.00	19847.952	0.243	.918
R	Medial area 7 (PEp)	6, −65, 51	0.00	19853.577	0.187	.946
L	Caudal lingual gyrus	−11, −82, −11	0.00	19853.239	0.254	.915
R	Caudal lingual gyrus	10, −85, −9	0.01	19850.278	3.073	.463
L	Caudal cuneus gyrus	−6, −94, 1	0.00	19842.742	0.848	.793
R	Caudal cuneus gyrus	8, −90, 12	0.00	19843.200	0.444	.838
L	Middle occipital gyrus	−31, −89, 11	0.00	19842.496	0.390	.863
R	Middle occipital gyrus	34, −86, 11	0.00	19846.381	0.730	.807
L	Area V5/MT+	−46, −74, 3	0.04	19844.622	8.334	.129
R	Area V5/MT+	48, −70, −1	0.00	19843.776	0.921	.792
L	Occipital polar cortex	−18, −99, 2	0.02	19848.965	4.604	.269
R	Occipital polar cortex	22, −97, 4	0.00	19841.741	0.140	.964
L	Inferior occipital gyrus	−30, −88, −12	0.00	19840.964	1.165	.738
R	Inferior occipital gyrus	32, −85, −12	0.01	19844.740	2.954	.471
L	Medial superior occipital gyrus	−11, −88, 31	0.00	19849.498	0.000	.999
R	Medial superior occipital gyrus	16, −85, 34	0.00	19845.463	0.024	.974
L	Lateral superior occipital gyrus	−22, −77, 36	0.00	19849.111	0.096	.967
R	Lateral superior occipital gyrus	29, −75, 36	0.00	19839.991	0.602	.838
L	Rostral hippocampus	−22, −14, −19	0.00	19846.880	0.173	.946
R	Rostral hippocampus	22, −12, −20	0.00	19847.074	0.256	.915
L	Caudal hippocampus	−28, −30, −10	0.00	19845.861	0.967	.774

**Table A4.** Memory Phase × Feature Type × Memory Success Null Results

*Hemisphere*	*Anatomical Label*	*MNI Coordinates*	*Sum of Squares*	*Denominator df*	*F*	*FDR-adjusted p*
L	Area 41/42	−54, −32, 12	0.00	19840.751	0.584	.838
L	TE1.0 and TE1.2	−50, −11, 1	0.00	19843.317	0.101	.967
R	TE1.0 and TE1.2	51, −4, −1	0.00	19843.464	0.843	.793
L	Caudal area 22	−62, −33, 7	0.00	19843.779	0.552	.838
R	Caudal area 22	66, −20, 6	0.00	19849.570	0.050	.967
L	Caudal area 21	−65, −30, −12	0.00	19849.383	0.732	.807
R	Caudal area 21	65, −29, −13	0.00	19850.720	0.462	.838
L	Dorsolateral area 37	−59, −58, 4	0.00	19845.615	0.003	.998
R	Dorsolateral area 37	60, −53, 3	0.00	19849.071	0.571	.838
L	Anterior superior temporal sulcus	−58, −20, −9	0.01	19842.199	2.300	.567
R	Anterior superior temporal sulcus	58, −16, −10	0.00	19843.447	0.490	.838
L	Extreme lateroventral area 37	−51, −57, −15	0.00	19846.319	0.788	.798
R	Extreme lateroventral area 37	53, −52, −18	0.00	19840.085	0.147	.963
L	Ventrolateral area 37	−55, −60, −6	0.00	19845.537	0.059	.967
R	Ventrolateral area 37	54, −57, −8	0.00	19846.909	0.062	.967
L	Caudolateral area 20	−59, −42, −16	0.01	19846.996	1.414	.717
R	Caudolateral area 20	61, −40, −17	0.00	19842.084	0.005	.998
L	Caudoventral area 20	−55, −31, −27	0.00	19846.206	1.156	.738
R	Caudoventral area 20	54, −31, −26	0.00	19847.155	0.236	.921
L	Medioventral area 37	−31, −64, −14	0.00	19845.552	0.540	.838
R	Medioventral area 37	31, −62, −14	0.00	19843.823	0.353	.875
L	Lateroventral area 37	−42, −51, −17	0.00	19843.705	0.022	.978
R	Lateroventral area 37	43, −49, −19	0.00	19844.490	0.013	.995
L	Rostroposterior superior temporal sulcus	−54, −40, 4	0.00	19845.896	0.013	.995
R	Rostroposterior superior temporal sulcus	53, −37, 3	0.00	19843.050	0.122	.967
L	Caudoposterior superior temporal sulcus	−52, −50, 11	0.01	19846.546	1.661	.696
R	Caudoposterior superior temporal sulcus	57, −40, 12	0.00	19852.056	0.280	.902
L	Caudal area 7	−15, −71, 52	0.00	19846.816	0.072	.967
R	Caudal area 7	19, −69, 54	0.00	19843.932	0.036	.967
L	Intraparietal area 7 (hIP3)	−27, −59, 54	0.00	19844.245	0.109	.967
R	Intraparietal area 7 (hIP3)	31, −54, 53	0.00	19844.881	0.018	.983
L	Caudal area 39 (PGp)	−34, −80, 29	0.00	19849.311	0.067	.967
R	Caudal area 39 (PGp)	45, −71, 20	0.01	19845.066	2.931	0.471
L	Rostrodorsal area 39 (Hip3)	−38, −61, 46	0.00	19850.419	1.207	.738
R	Rostrodorsal area 39 (Hip3)	39, −65, 44	0.00	19842.794	0.005	.998
L	Rostrodorsal area 40 (PFt)	−51, −33, 42	0.00	19848.338	0.548	.838
R	Rostrodorsal area 40 (PFt)	47, −35, 45	0.00	19849.142	0.048	.967
L	Caudal area 40 (PFm)	−56, −49, 38	0.00	19848.992	0.000	.999
R	Caudal area 40 (PFm)	57, −44, 38	0.00	19847.018	0.050	.967
L	Rostroventral area 39 (PGa)	−47, −65, 26	0.00	19846.334	0.041	.967
R	Rostroventral area 39 (PGa)	53, −54, 25	0.00	19850.448	0.009	.998
L	Rostroventral area 40 (PFop)	−53, −31, 23	0.00	19837.902	0.213	.938
R	Rostroventral area 40 (PFop)	55, −26, 26	0.00	19847.554	0.287	.898
L	Medial area 7 (PEp)	−5, −63, 51	0.00	19847.952	0.053	.967
R	Medial area 7 (PEp)	6, −65, 51	0.00	19853.577	0.459	.838
L	Caudal lingual gyrus	−11, −82, −11	0.00	19853.239	0.601	.838
R	Caudal lingual gyrus	10, −85, −9	0.00	19850.278	0.361	.875
L	Caudal cuneus gyrus	−6, −94, 1	0.00	19842.742	0.060	.967
R	Caudal cuneus gyrus	8, −90, 12	0.00	19843.200	0.081	.967
L	Middle occipital gyrus	−31, −89, 11	0.01	19842.496	1.279	.738
R	Middle occipital gyrus	34, −86, 11	0.01	19846.381	2.100	.581
L	Area V5/MT+	−46, −74, 3	0.01	19844.622	1.414	.717
R	Area V5/MT+	48, −70, −1	0.00	19843.776	0.847	.793
L	Occipital polar cortex	−18, −99, 2	0.01	19848.965	1.397	.717
R	Occipital polar cortex	22, −97, 4	0.02	19841.741	6.031	.211
L	Inferior occipital gyrus	−30, −88, −12	0.00	19840.964	0.111	.967
R	Inferior occipital gyrus	32, −85, −12	0.01	19844.740	1.676	.696
L	Medial superior occipital gyrus	−11, −88, 31	0.00	19849.498	0.857	.793
R	Medial superior occipital gyrus	16, −85, 34	0.01	19845.463	2.167	.579
L	Lateral superior occipital gyrus	−22, −77, 36	0.00	19849.111	0.003	.998
R	Lateral superior occipital gyrus	29, −75, 36	0.00	19839.991	1.081	.752
L	Rostral hippocampus	−22, −14, −19	0.00	19846.880	0.000	.999
R	Rostral hippocampus	22, −12, −20	0.00	19847.074	0.452	.838
L	Caudal hippocampus	−28, −30, −10	0.00	19845.861	0.056	.967

## Acknowledgments

We thank Lamont Conyers and Jennifer Graves for their extensive MRI support and all participants for their participation.

Corresponding author: Cortney M. Howard, Duke University, Levine Science Research Center, 308 Research Dr Suite M051, Durham, NC 27710, or via e-mail: cortney.howard@duke.edu.

## Data Availability Statement

All de-identified imaging and behavioral data will be shared upon request via e-mail to the corresponding author, Cortney Howard, or the principal investigator Simon Davis.

## Author Contributions

Cortney M. Howard: Conceptualization; Formal analysis; Methodology; Writing—Original draft. Shenyang Huang: Formal analysis; Methodology; Writing—Review & editing. Mariam Hovhannisyan: Data curation; Methodology. Roberto Cabeza: Funding acquisition; Supervision; Writing—Review & editing. Simon W. Davis: Conceptualization; Formal analysis; Funding acquisition; Methodology; Supervision; Writing—Review & editing.

## Funding Information

This study was supported by the National Institute of Health (https://dx.doi.org/10.13039/100000049), grant numbers: R01-AG066901 and K01-AG053539, and the National Science Foundation (https://dx.doi.org/10.13039/100023581), grant number: GRFP-DGE 2139754.

## Diversity in Citation Practices

Retrospective analysis of the citations in every article published in this journal from 2010 to 2021 reveals a persistent pattern of gender imbalance: Although the proportions of authorship teams (categorized by estimated gender identification of first author/last author) publishing in the *Journal of Cognitive Neuroscience* (*JoCN*) during this period were M(an)/M = .407, W(oman)/M = .32, M/W = .115, and W/W = .159, the comparable proportions for the articles that these authorship teams cited were M/M = .549, W/M = .257, M/W = .109, and W/W = .085 (Postle and Fulvio, *JoCN*, 34:1, pp. 1–3). Consequently, *JoCN* encourages all authors to consider gender balance explicitly when selecting which articles to cite and gives them the opportunity to report their article's gender citation balance.
